# Survival-Convolution Models for Predicting COVID-19 Cases and Assessing Effects of Mitigation Strategies

**DOI:** 10.3389/fpubh.2020.00325

**Published:** 2020-07-03

**Authors:** Qinxia Wang, Shanghong Xie, Yuanjia Wang, Donglin Zeng

**Affiliations:** ^1^Department of Biostatistics, Mailman School of Public Health, Columbia University, New York, NY, United States; ^2^Department of Biostatistics, Gillings School of Public Health, University of North Carolina at Chapel Hill, Chapel Hill, NC, United States

**Keywords:** COVID-19, survival-convolution model, time-varying effective reproduction number, mitigation measures, prediction

## Abstract

Countries around the globe have implemented unprecedented measures to mitigate the coronavirus disease 2019 (COVID-19) pandemic. We aim to predict the COVID-19 disease course and compare the effectiveness of mitigation measures across countries to inform policy decision making using a robust and parsimonious survival-convolution model. We account for transmission during a pre-symptomatic incubation period and use a time-varying effective reproduction number (*R*_*t*_) to reflect the temporal trend of transmission and change in response to a public health intervention. We estimate the intervention effect on reducing the transmission rate using a natural experiment design and quantify uncertainty by permutation. In China and South Korea, we predicted the entire disease epidemic using only early phase data (2–3 weeks after the outbreak). A fast rate of decline in *R*_*t*_ was observed, and adopting mitigation strategies early in the epidemic was effective in reducing the transmission rate in these two countries. The nationwide lockdown in Italy did not accelerate the speed at which the transmission rate decreases. In the United States, *R*_*t*_ significantly decreased during a 2-week period after the declaration of national emergency, but it declined at a much slower rate afterwards. If the trend continues after May 1, COVID-19 may be controlled by late July. However, a loss of temporal effect (e.g., due to relaxing mitigation measures after May 1) could lead to a long delay in controlling the epidemic (mid-November with fewer than 100 daily cases) and a total of more than 2 million cases.

## 1. Introduction

The COVID-19 pandemic is currently a daunting global health challenge. The novel coronavirus was observed to have a long incubation period and highly infectious during this period (1–4). The cumulative case number surpasses 4.1 million by May 10, with more than 1.3 million in the United States (US). It is imperative to study the course of the disease outbreak in countries that have controlled the outbreak (e.g., China and South Korea) and compare mitigation strategies to inform decision making in regions that are in the midst of (e.g., the US) or at the beginning of outbreak (e.g., South America).

Various infectious disease models (5–7) are proposed to estimate the transmission of COVID-19 (8–12) and investigate the impact of public health interventions on mitigating the spread (13–17). Several studies modeled the transmission by stochastic dynamical systems (8–10, 15), such as susceptible-exposed-infectious-recovered (SEIR) models (8), extended Kalman filter (18–20), and individual-based simulation models (13, 14). Some models did not explicitly take into account of behavioral change (e.g., social distancing) and government mitigation strategies that can have major influences on the disease course, while other work modified the transmission rate as public-health-intervention-dependent (15, 17) or time-varying (10). A recent study (16) considered the disease incubation period and used a convolution model based on SEIR. A state-space susceptible-infectious-recovered (SIR) model with time-varying transmission rate (21) was developed to account for interventions and quarantines.

SEIR models can incorporate mechanistic characteristics and scientific knowledge of virus transmission to provide useful estimates of its temporal dynamics, especially when individual-level epidemiological data are available through surveillance and contact tracing. However, these sophisticated models may involve a large number of parameters and assumptions about individual transmission dynamics. They may thus be susceptible to perturbation of parameters and prior assumptions, yielding wide confidence intervals especially when granular individual-level data are not available. In contrast to infectious disease models, alternative statistical models are proposed to predict summary statistics such as deaths and hospital demand under a non-linear mixed effects model framework (22), survival analysis has been introduced to model the occurrence of clinical events in infectious disease studies (23), and a non-parametric space-time transmission model was developed to incorporate spatial and temporal information for predictions at the county level (24). Non-parametric modeling or survival models are data-driven, and parameters may therefore not be scientifically related to disease epidemic.

In this work, we propose a parsimonious and robust population-level survival-convolution model that is based on main characteristics of COVID-19 epidemic and observed number of confirmed cases to predict disease course and assess public health intervention effect. Our method models only key statistics (e.g., daily new cases) that reflect the disease epidemic over time with at most six parameters, and it may therefore be more robust than models that rely on individual transmission processes or a large number of parameters and assumptions. We constructed our model based on prior scientific knowledge about COVID-19 instead of *post-hoc* observations of the trend of disease spread. Specifically, three important facts we consider include that (1) SARS-CoV-2 virus has an incubation period up to 14–21 days (1), and a patient can be highly infectious in the pre-symptomatic phase; (2) the transmission rate varies over time and can change significantly when government guidelines and mitigation strategies are implemented; and (3) the intervention effect may be time-varying.

We aim to achieve the following goals. The first goal is to fit observed data to predict daily new confirmed cases and latent pre-symptomatic cases, the peak date, and the final total number of cases. The second goal is to assess the effect of nationwide major interventions across countries (e.g., mitigation measures) under the framework of natural experiments [e.g., longitudinal pre-post quasi-experimental design, (25)]. Quasi-experiment approaches are often used to estimate intervention effect of a public health intervention [e.g., HPV vaccine, (26)] or a health policy where randomized controlled trials (RCTs) are not feasible. Our third goal is to project the future trend of COVID-19 for the countries (e.g., US) amid the epidemic under different assumptions of future transmission rates, including the continuation of the current trend and relaxing mitigation measures.

## 2. Methods

### 2.1. Data Source

We used data from a publicly available database that consolidates multiple sources of official reports (World Meters[https://www.worldometers.info/coronavirus/]). We analyzed two countries with a large number of confirmed cases in Asia (China and South Korea) and two outside (Italy and US). Since both China and South Korea are already at the end of epidemic, we used their data to test empirical prediction performance of our method. We included data in the early phase of epidemic as training set to estimate model parameters and leave the rest of the data as testing set for evaluation. For China, we used data up to 2 weeks post the lockdown of Wuhan city (January 23) as training (data from January 20 to February 4), and we used the remaining observed data for evaluation (February 5 to May 10). Similarly, for South Korea we used data from February 15 to March 4 as training and leave the rest for evaluation (March 5 to May 10). Italy is the first European country confronted by a large outbreak and currently has passed its peak. We estimated the effect of the nation-wide lockdown in Italy (dated March 11) using 10 weeks data (February 20 to April 29). For the US, as, after May 1, some mitigation measures were lifted in various states, we also included about 10 weeks data (February 21 to May 1) to assess the effect of its mitigation strategies.

### 2.2. Survival-Convolution Model

Let *t* denote the calendar time (in days) and let *N*_0_(*t*) be the number of individuals who are newly infected by COVID-19 at time *t*. Let *t*_*j*_ denote the time when individual *j* is infected (*t*_*j*_ = ∞ if never infected), and let *T*_*j*_ be the duration of this individual remaining infectious to any other individual and in the transmission chain. Let *t*_0_ be the unknown calendar time when the first patient (patient zero) is infected. Therefore, at time *t*, the total number of individuals who can infect others is $\underset{j}{\sum }I\left(t_{j}\leq t,T_{j}\geq t-t_{j}\right)=\overset{C}{\underset{m=0}{\sum }}\underset{\left\{j\text{:}j\text{is infected at}\left(t-m\right)\right\}}{\sum }I\left(T_{j}\geq m\right),$where *C* = min(*t* − *t*_0_, *C*_1_) with *C*_1_ as the maximum incubation period (i.e., 21 days for SARS-CoV-2) and *I*(*E*) denotes an indicator function with *I*(*E*) = 1 if event *E* occurs and *I*(*E*) = 0 otherwise. Since the total number of individuals who are newly infected at time (*t* − *m*) is *N*_0_(*t* − *m*), the number of individuals who remain infectious at time *t* is $M\left(t\right)=\overset{C}{\underset{m=0}{\sum }}N_{0}\left(t-m\right)S\left(m\right),$ where *S*(*m*) denotes the proportion of individuals remaining infectious after *m* days of being infected, or, equivalently, the survival probability at day *m* for *T*_*j*_. On the other hand, right after time *t*, some individuals will no longer be in the transmission chain (e.g., due to testing positive and quarantine or out of infectious period) with duration *T*_*j*_ = (*t* − *t*_*j*_). The total number of these individuals is $\underset{j}{\sum }I\left(t_{j}\leq t,T_{j}=t-t_{j}\right)=\overset{C}{\underset{m=0}{\sum }}\underset{j\text{:}j\text{is infected at}\left(t-m\right)}{\sum }I\left(T_{j}=m\right)$, or equivalently

(1) $$\begin{array}{l} Y\left(t\right)=\overset{C}{\underset{m=0}{\sum }}N_{0}\left(t-m\right)\left[S\left(m\right)-S\left(m+1\right)\right]. \end{array}$$

Therefore, (*M*(*t*) − *Y*(*t*)) is the number of individuals who can still infect others after time *t*. Assuming the transmission rate at *t* to be *a*(*t*), at time (*t* + 1), the number of newly infected patients is *a*(*t*)[*M*(*t*) − *Y*(*t*)], which yields

(2) $$\begin{array}{l} N_{0}\left(t+1\right)=a\left(t\right)\overset{C}{\underset{m=0}{\sum }}N_{0}\left(t-m\right)S\left(m+1\right). \end{array}$$

Note that *a*(*t*) is time-varying because the transmission rate depends on how many close contacts an infected individual may have at time *t*, which is affected by public heath interventions (e.g., stay-at-home order, lockdown), and saturation level of the infection in the whole population. Define $R_{t}=\overset{C}{\underset{m=0}{\sum }}a\left(t+m\right)S\left(m\right)$, the expected number of secondary cases infected by a primary infected individual in a population at time *t* while accounting for the entire incubation period of the primary case. Thus, *R*_*t*_ is the instantaneous time-varying effective reproduction number (27) that measures temporal changes in the disease spread.

Models (1) and (2) provide a robust dynamic model to characterize COVID-19 epidemic. Equation (2) gives a convolution update for the new cases using the past numbers, while equation (1) gives the number of cases out of transmission chain at time *t*, and *M*(*t*) computes the number of latent pre-symptomatic cases by the end of time *t*. This model considers three important quantities to characterize COVID-19 transmission: the initial date, *t*_0_, of the first (likely undetected) case in the epidemic, the survival function of time to out of transmission, *S*(*m*), and the transmission rate over calendar time, *a*(*t*).

We model the transmission rate *a*(*t*) as a non-negative, piece-wise linear function with knots placed at meaningful event times. The simplest model consists of a constant and a single linear function with three parameters [infection date of patient zero and the intercept and slope of *a*(*t*)]. When a massive public health intervention (e.g., nation-wide lockdown) is implemented at some particular date, we introduce an additional linear function afterwards with a new slope parameter. Thus, the difference in slope parameters of *a*(*t*) before and after an intervention reflects its effect on reducing the rate of change in disease transmission (i.e., “flattening the curve”). Since the intervention effect may diminish over time, we introduce another slope parameter 2 weeks after intervention to capture the longer-term effect. We use existing knowledge of the SARS-CoV-2 virus incubation period (1) to approximate *S*(*m*) and perform sensitivity analysis assuming different parameters. For estimation, we minimize a loss function measuring differences between model predicted and observed daily number of cases. For statistical inference, we use permutation based on standardized residuals. All mathematical details are in Supplementary Material.

### 2.3. Utility of Our Model

First, with parameters estimated from data and assuming that the future transmission rate remains the same trend, we can use models (1) and (2) to predict future daily new cases, the peak time, expected number of cases at the peak, when *R*_*t*_ will be reduced to below 1.0 and the epidemic will be controlled (the number of daily new cases below a threshold or decreases to zero). Furthermore, our model provides the number of latent cases cumulative over the incubation period at each future date, which can be useful to anticipate challenges and allocate resources effectively.

Second, we can estimate the effects of mitigation strategies, leveraging the nature of quasi-experiments where subjects receive different interventions before and after the initiation of the intervention. The longitudinal pre-post intervention design allows valid inferences, assuming that pre-intervention disease trend would have continued had the intervention not taken place and local randomization holds (whether a subject falls immediately before or after the initiation date of an intervention may be considered as random, and the “intervention assignment” may thus be considered to be random). Applying this design, the intervention effects will be estimated as the difference in the rate of change of the transmission rate function before and after an intervention takes place.

Third, we can study the impact of an intervention (e.g., lifting mitigation measures) that changes the epidemic at a future date. Using permutations, we can obtain the joint distribution of the parameter estimators and construct confidence intervals (CI) for the projected case numbers and interventions effects.

## 3. Results

**Figure 1:**
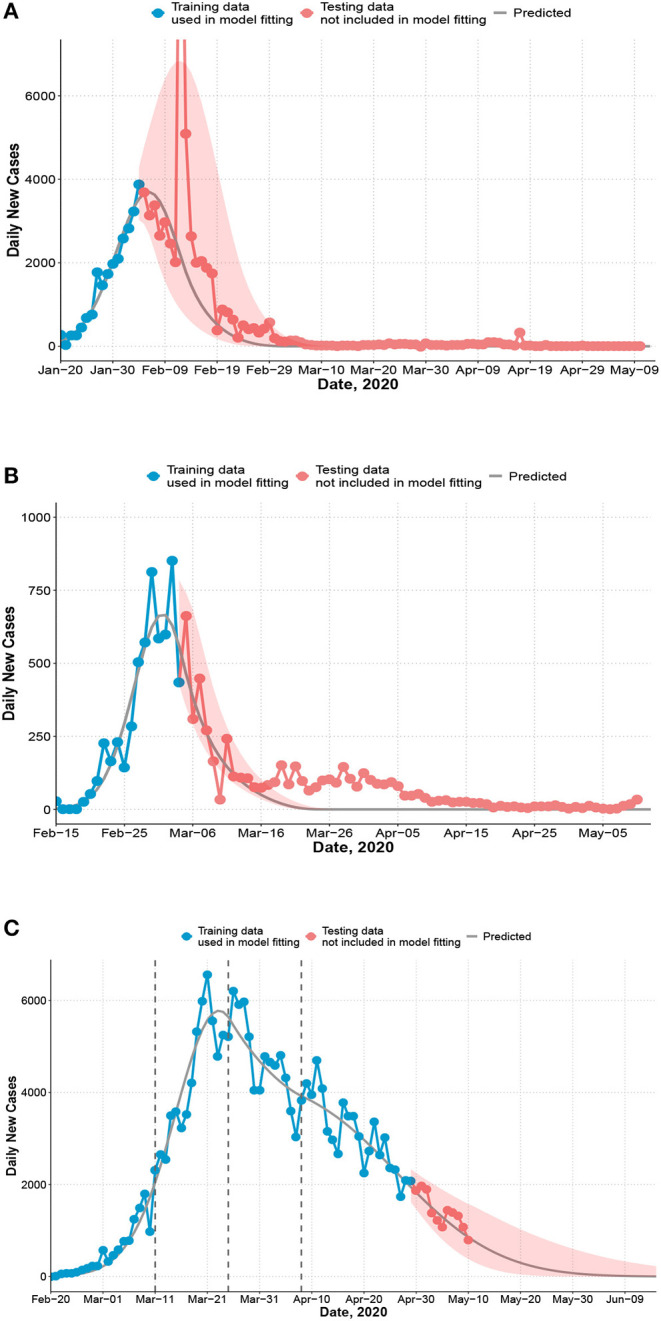
Observed and predicted daily new cases and 95% confidence interval (shaded). **(A)** China. Training data: January 20 to February 4; testing data: February 5 to May 10. 14,108 cases were reported on February 12 and not shown on figure. The recent cases since April are imported cases. **(B)** South Korea. Training data: February 15 to March 4; testing data: March 5 to May 10. **(C)** Italy. First dashed line indicates the nation-wide lockdown (March 11). Second and third dashed line indicates 2 or 4 weeks after. Training data: February 20 to April 29 (7 weeks after the lockdown); testing data: April 30 to May 10.

**Figure 2:**
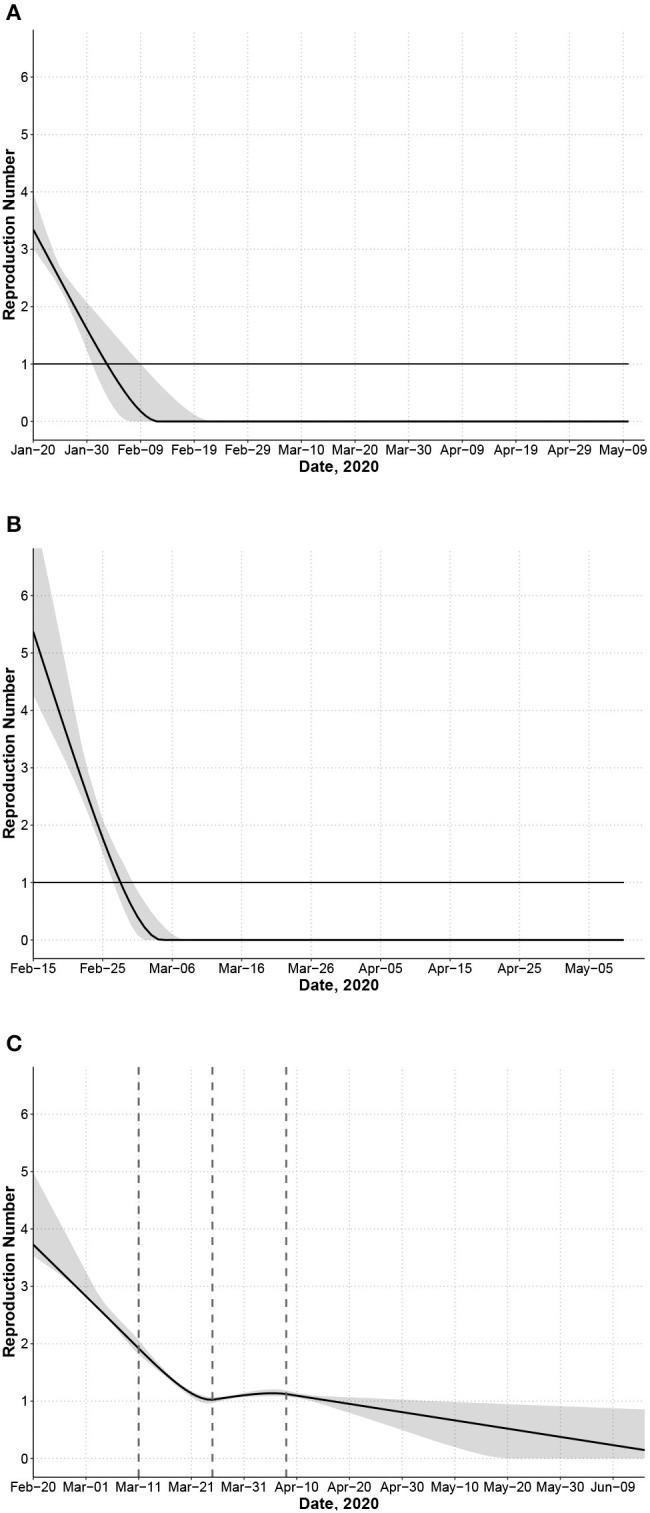
Effective reproduction number *R*_*t*_ for each country computed as the average number of secondary infections generated by a primary case at time *t* accounting for the incubation period of the primary case. Dashed lines indicate knots for transmission rate *a*(*t*). **(A)** China. **(B)** South Korea. **(C)** Italy.

For China, the transmission rate *a*(*t*) is a single linear function (estimates in Table 1). The first community infection was estimated to occur on January 3, 17 days before the first reported case (Table 1). Figure 1A shows that the model captures the peak date of new cases, the epidemic end date, and the confidence interval contains the majority of observed number of cases except one outlier (due to a change of diagnostic criteria). The reproduction number *R*_*t*_ decreases quickly from 3.34 to below 1.0 in 14 days (Figure 2A). We only used data up to February 4 to estimate our model. The observed total number of cases by May 10 is 82,901, which is inside the 95% CI of the estimated total number of cases [58,415; 95% CI: (42,516, 133,083)]. There are two outlier days (February 12, 13) with a total of 19,198 cases reported in the testing set. Excluding two outliers, the observed number of cases 62,356.

**Table 1:** Model estimated parameters in each country.

**Country**	**Parameter**	**Estimate**	**95% CI**
	or prediction^*^		
China	*t*_0_(*d*)	Jan 3 (17)	(12, 21)^**^
Training data: Jan 20 to Feb 4	*a*_0_	0.793	(0.68, 1.02)
Testing data: Feb 5 to May 10	*a*_1_	-0.693	(-1.13, -0.42)
	Duration	44	(39, 55)
	End date	Mar 4	(Feb 28, Mar 15)
	Total	58,415	(42,516, 133,083)
South Korea	*t*_0_(*d*)	Feb 11 (4)	(1, 7)
Training data: Feb 15 to Mar 4	*a*_0_	1.363	(1.03, 1.98)
Testing data: Mar 5 to May 10	*a*_1_	-1.496	(-2.39, -0.96)
	Duration	39	(37, 43)
	End date	Mar 25	(Mar 23, Mar 29)
	Total	7,977	(7,307, 10,562)
Italy	*t*_0_(*d*)	Feb 10 (10)	(4, 11)
Training data: Feb 20 to Apr 29	*a*_0_	0.789	(0.73, 1.10)
Testing data: Apr 30 to May 10	*a*_1_	-0.358	(-0.68, -0.26)
	*a*_2_	-0.372	(-0.46, -0.31)
	*a*_3_	0.061	(0.02, 0.12)
	*a*_4_	-0.057	(-0.12, -0.01)
	Duration	123	(103, 179)
	End date	Jun 22	(Jun 2, Aug 17)
	Total	223,410	(216,848, 257,710)
United States	*t*_0_(*d*)	Feb 15 (6)	(1, 4)
Training data: Feb 21 to May 1	*a*_0_	0.410	(0.34, 0.62)
Testing data: May 2 to May 10	*a*_1_	0.526	(0.23, 0.72)
	*a*_2_	-1.031	(-1.24, -0.86)
	*a*_3_	-0.042	(-0.06, -0.03)
Scenario 1: Continue current^†^	Duration	156	(139, 188)
	End date	Jul 26	(Jul 9, Aug 27)
	Total	1,626,950	(1,501,036, 1,918,602)
Scenario 2: 50% slower	Duration	188	(163, 233)
after May 1	End date	Aug 27	(Aug 2, Oct 11)
	Total	1,731,992	(1,563,122, 2,113,294)
Scenario 3: 75% slower	Duration	226	(190, 289)
after May 1	End date	Oct 4	(Aug 29, Dec 5)
	Total	1,832,291	(1,616,574, 2,324,552)
Scenario 4: 100% slower	Duration^‡^	272	(201, 448)
after May 1	Control date^‡^	Nov 19	[Sep 9, May 13 (2021)]
	Total^‡^	2,084,235	(1,728,028, 3,094,518)

* t_0_ is the estimated date of the first undetected community infection; d is the estimated gap days between the first undetected case and the first reported case; a_0_ is the transmission rate before the reported first case; a_1_, a_2_, and a_3_ are rates of change of a(t) in each period measured as change per 21 days; “Duration” is the number of days from the date of the first reported case to “End date”; “End date” is the date when predicted new case decreases to zero; and “Total” is the total number of predicted cases by the “End date.”

** CI for d.

† Scenario 1 assumes the transmission rate decreases at the same rate (i.e., a_3_) after May 1; Scenarios 2–4 assume the relaxation of quarantine measures after May 1 will lead to a slower decrease of transmission rate by 50, 75, and 100% (complete loss of temporal effect over time).

‡ *Under scenario 4, “Duration” and “Control date” is defined by the date when the predicted daily new case is less than 100 since the distribution of new cases has an extremely long tail (the end date defined by zero new case is May 3, 2021; CI: Dec 27, 2021 to Mar 16, 2022); and “Total” is the total predicted cases by the “Control date”*.

For South Korea, Figure 1B shows that the model captures the general trend of the epidemic except at the tail area (after March 15) where some small and enduring outbreak is observed. The effective reproduction number decreases dramatically from 5.37 at the beginning of the outbreak to below 1.0 in 14 days (Figure 2B). The predicted number of new cases at the peak is 665 and the total number of predicted cases at the peak time is close to the observed total (4,300 vs. 4,335). The predicted total number by March 15 is 7,816 and the observed total is 8,162.

For Italy, we model *a*(*t*) as a four-piece linear function to account for the change in mitigation strategies with a knot placed at the lockdown (March 11), and two additional knots at 2-week intervals (March 25, April 8) to account for a time-varying intervention effect (during the immediate 2 weeks, next 2 weeks, and afterwards). The difference in the rate of change before and after the first knot measures the immediate effect of lockdown on reducing the transmission rate. Change before and after the second and third knot measures whether the lockdown effect can be maintained in longer term. The rate of change in *R*_*t*_ is not significantly different before and 2 weeks after the lockdown (Figure 2C). The reproduction number decreased from 3.73 at the beginning to 1.02 2 weeks post-lockdown. However, starting from the third week post-lockdown (March 26), *R*_*t*_ stops decreasing and remains close to 1.0 until April 16. The slope of *a*(*t*) increases by 116% to a slightly positive value after March 26 (Table 1, comparing *a*_2_ and *a*_3_ for Italy). This is consistent with a relatively flat trend of observed daily new cases during this period (Figure 1C). The estimated total by May 10 is 216,300 [95%CI: (214,863, 228,406)] and close to the observed total (219,070). Recent daily cases in the testing set also closely follow our predicted trend (Figure 1C).

**Figure 3:**
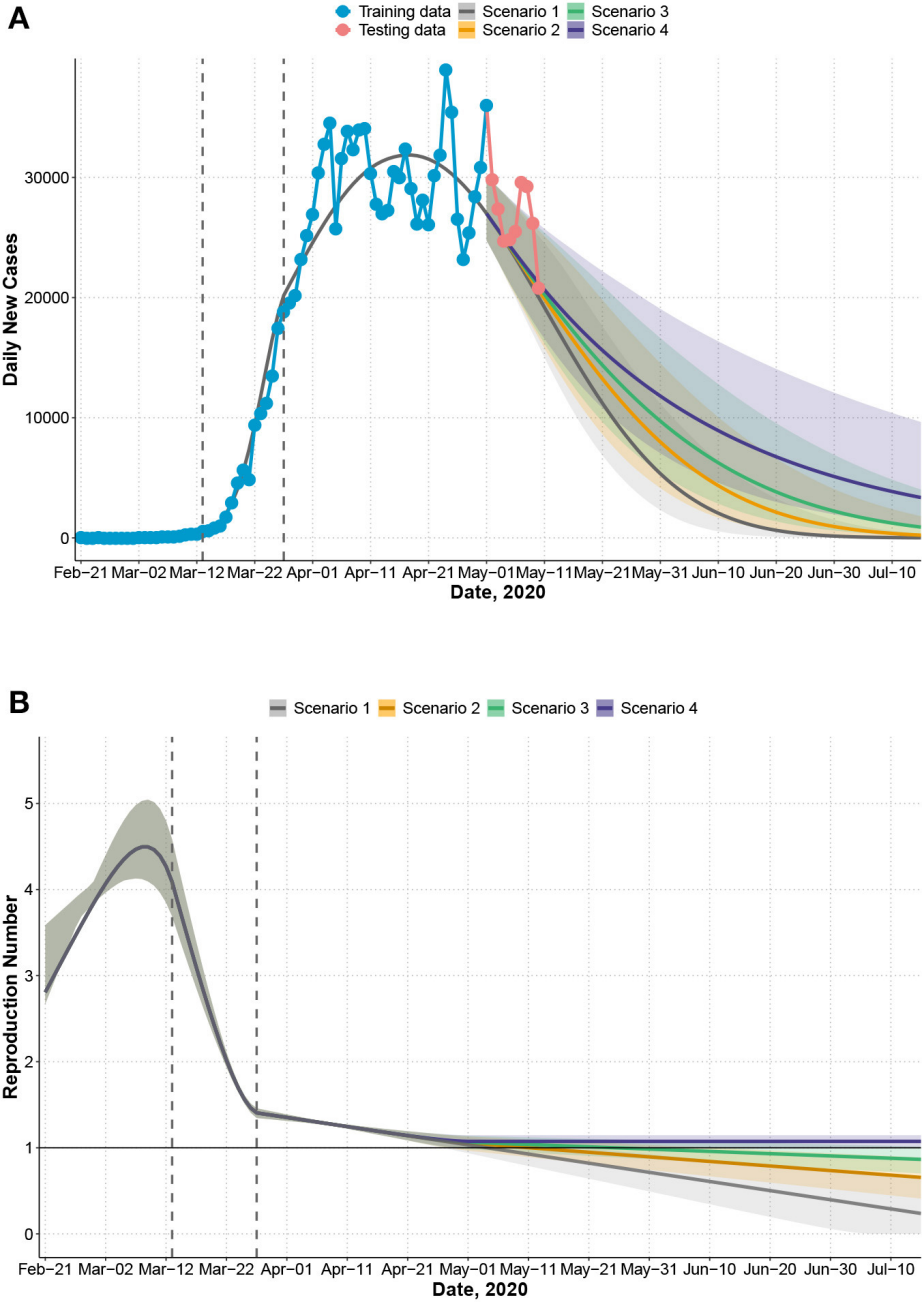
United States: observed and predicted daily new cases, 95% confidence intervals under four scenarios that assume relaxation of mitigation measures occurs after May 1. Scenario 1: transmission rate *a*(*t*) follows the same trend after May 1 as observed between March 27 and May 1. Scenario 2: rate of decrease of *a*(*t*) slows by 50% after May 1. Scenario 3: rate of decrease of *a*(*t*) slows by 75% after May 1. Scenario 4: rate of decrease of *a*(*t*) slows by 100% after May 1 (complete loss of temporal decreasing effect). First dashed line indicates the declaration of national emergency (March 13). Second dashed line indicates 2 weeks after (March 27). Training data: February 21 to May 1 (7 weeks after declaring national emergency); testing data: May 2 to May 10. **(A)** Observed and predicted daily new cases. **(B)** Effective reproduction number *R*_*t*_.

In the US, we fit a three-piece model for *a*(*t*) with a knot on March 13 (the declaration of national emergency) and an additional knot 2 weeks after (March 27) to account for potential changes in the transmission rate. The predicted peak date is May 3 (Figure 3A) with a total number of 1,176,915 cases by May 3, which is close to the observed total (1,188,122). *R*_*t*_ increases during the early phase but decreases sharply after the declaration of national emergency (Figure 3B) up to 2 weeks after. During the next period (March 28 to April 10), *R*_*t*_ decreases at a much slower rate. If this trend continues, the end of epidemic date is predicted to be July 26 (scenario 1, Figure 3A, Table 1). However, since states in the US are gradually lifting mitigation measures after May 1, the trend of transmission rate may change. We predicted the epidemic control date, assuming *a*(*t*) decreases slower after May 1 by 50% (scenario 2), 75% (scenario 3), and 100% (scenario 4) in Table 1. Under scenario 4, where the temporal effect of mitigation measures is completely lost [i.e., *a*(*t*) is a constant over time], the projected total number of cases will be more than 2 million, and the epidemic cannot be controlled until November 19 (with less than 100 daily cases Table 1). We provide an updated analysis of the US epidemic with more training data until May 29 (Supplementary Material). The predicted recent trend is closer to scenario 4 with a control date in November and 2.7 million total cases. Assuming a case fatality rate of 6% as observed by May 10, the total number of deaths would be around 162,000 by November.

The estimated number of latent cases present on each day (i.e., including pre-symptomatic patients infected *k* days before but have not shown symptoms) can be seen in the Supplementary Material (Figure S1). For all countries, there were a large number of latent cases around the peak time. We performed a sensitivity analysis using different distributions of *S*(*m*) assuming a delay in reporting confirmed cases. The results show that predicted daily new cases were similar under different parameters of *S*(*m*) for both US and Italy (Figures S2, S3), demonstrating robustness of our method to the assumptions of *S*(*m*).

## 4. Discussion

In this study, we propose a parsimonious and robust survival convolution model to predict daily new cases of the COVID-19 outbreak and use a natural quasi-experimental design to estimate the effects of mitigation measures. Our model accounts for major characteristics of COVID-19 (long incubation period and highly contagious during incubation) with a small number of parameters (up to six) and assumptions, directly targets prediction accuracy, and provides measures of uncertainty and inference based on permuting the residuals. We allow the transmission rate to depend on time and modify the basic reproduction number *R*_0_ as a time-dependent measure *R*_*t*_ to estimate change in disease transmission over time. Thus, *R*_*t*_ corrects for the naturally impact of time on the disease spread. Our estimated reproduction number at the beginning of the epidemic ranges from 2.81 to 5.37, which is consistent with the *R*_0_ reported in other studies (28) (range from 1.40 to 6.49 with a median of 2.79). For predicting daily new cases, our analyses suggest that the model estimated from early periods of outbreak can be used to predict the entire epidemic if the disease transmission rate dynamic does not change dramatically over the disease course (e.g., about 2 weeks of data is sufficient for China and fits the general trend of South Korea).

Comparing the effective reproduction numbers across countries, *R*_*t*_ decreased much more rapidly in South Korea and China than Italy (Figure 1). In South Korea, the effective reproduction number had been reduced from 5.37 to under 1.0 in a mere 13 days, and the total number of cases is low. The starting reproduction number in South Korea was high possibly due to many cases linked to patient 31 and outbreaks at church gatherings. Similarly, for China, the reproduction number reduced to below 1.0 in 14 days. Italy's *R*_*t*_ decreased until almost reaching 1.0 on March 25 but remained around 1.0 for 3 weeks. The US followed a fast decreasing trend during a 2-week period after declaring national emergency (*a*_2_ = −1.031), which is faster than the first 2 weeks in China (*a*_1_ = −0.693), but its *R*_*t*_ decreased at a much slower rate (*a*_3_ = −0.042) afterwards and was below 1.0 on May 5.

Comparing mitigation strategies across countries, the fast decline in *R*_*t*_ in China suggests that the initial mitigation measures put forth on January 23 (lockdown of Wuhan city, traffic suspension, home quarantine) were successful in controlling the transmission speed of COVID-19. Additional mitigation measures were in place after February 2 (centralized quarantine and treatment) but did not seem to have significantly changed the disease course. In fact, our model assumes the same transmission rate trajectory after February 2 fits all observed data up to May 10. A recent analysis of Wuhan's data (29, 30) arrived at a similar conclusion, and their estimated *R*_*t*_ closely matches with our estimates. However, their analyses were based on self-reported symptom onset and other additional surveillance data, where we used only widely available official reports of confirmed cases. Another mechanistic (31) study confirmed the effectiveness of early containment strategies in Wuhan.

South Korea did not impose a nation-wide lockdown or closure of businesses but, at the very early stage (when many cases linked to patient 31 were reported on February 20), conducted extensive broad-based testing and detection (drive through tests started on February 26), rigorous contact tracing, isolation of cases, and mobile phone tracking. Our results suggest that South Korea's early mitigation measures were also effective.

Italy's initial mitigation strategies in the most affected areas reduced *R*_*t*_ from 3.73 to 1.92 in 20 days. To estimate the effect of the nation-wide lockdown as in a natural experiment, we require local randomization and the continuity assumption. The former requires that characteristics of subjects who are infected right before or after the lockdown are similar. Since, in a very short time period, whether a person is infected at time *t* or *t*+1 is likely to be random, local randomization is likely to be valid. Continuity assumption refers to that the transmission rate before the lockdown would be the same as the trend afterwards had the intervention not been implemented. Under this assumption, the lockdown in Italy is not effective in further reducing the transmission speed [slopes of *a*(*t*) are similar before and after lockdown on March 11]. There were 10,149 cases reported in Italy as of March 10, suggesting that the lockdown was placed after the wide community spread had already occurred. Nevertheless, it is possible that without the lockdown the transmission rate would have had increased, i.e., the lockdown enhanced and maintained the effect of quarantine for 2 weeks. In fact, after 2 weeks of lockdown, we observe a loss of temporal effect so that *R*_*t*_ has remained around 1.0 for about 2–3 weeks before it starts to decrease again.

For the US, *R*_*t*_ was as high as 4.50 before the declaration of national emergency on March 13 but declines rapidly over a 2-week period after March 13. Although the disease trend and mitigation strategies vary across states in the US, since the declaration of national emergency, many states have implemented social distancing and ban of large gathering. The large difference before and 2 weeks after March 13 is likely due to states with large numbers of cases that implemented state-wide stay-at-home orders (e.g., New York and New Jersey), which indicates that these measures may be effective. Our model estimated a continued decrease in *R*_*t*_ from March 27 to May 1 but at a much slower rate ( 95.9% slower; Table 1, comparing *a*_2_ and *a*_3_ for the US) when it approached 1.0. In China, centralized quarantine and treatment were implemented when *R*_*t*_ was around 1.0 (29), which assisted in quick further reduction of *R*_*t*_ to zero and final control of the epidemic. If the trend in US continues after May 1, the first wave of epidemic will be controlled by July 26 (CI: July 9, August 27). However, after May 1, many states enter a re-opening phase. If the guidelines on quarantine measures are relaxed in order for the temporal effect of quarantine measures to be completely lost, the predicted total number of cases is more than 2 million, with a long delay in controlling the epidemic (less than 100 cases by November 19 and no new case by May, 2021). In an updated analysis that includes additional observed data in May, the recent *R*_*t*_ is near a constant between 1.1 and 1.2 from April 11 to May 29, and the confidence interval suggests some possibility of an uptake of new cases (Supplementary Material). These results suggest that the epidemic in the US is still not yet fully under control by June 7, especially in certain states that present a consistent increase of daily new cases since re-opening. Careful mitigation measures should be maintained to prevent an uptake in daily new cases and another outbreak. These prediction results will be regularly updated at our Github website (https://github.com/COVID19BIOSTAT/covid19_prediction).

Other studies reported transmission between asymptomatic individuals (9), which is not accounted for here. However, asymptomatic individuals can only be identified and confirmed by serological tests which are not widely available. When there is a delay in reporting some symptomatic patients, the daily reported cases are a mixture of new symptomatic cases and patients presenting after having had symptoms for a few days. In this case, the average number of days to testing positive may be higher than the virus incubation period of 5.2 days. However, as shown in our sensitivity analysis, the prediction of daily reported cases was not affected by using a larger mean value for *S*(*m*), demonstrating robustness of the model. Our model does not consider subject-specific covariates and focuses on predicting population-level quantities. Neither have we considered borrowing information from multiple countries or state-level analysis for the US, which are worthy of study in a mixed effects model framework. We do not consider prediction of daily new deaths or hospitalizations. These data can be included to enhance the prediction of new cases by linking the distribution of time to COVID symptom onsets, hospitalization, or death. Lastly, we can consider a broader class of models for transmission rate *a*(*t*) to allow discontinuity in both intercepts and slopes before and after an intervention under a regression discontinuity design (26, 32).

Despite these limitations, our study offers several implications. Implementing mitigation measures earlier in the disease epidemic reduces the disease transmission rate at a faster speed (South Korea, China). Consequently, for regions at the early stage of disease epidemic, mitigation measures should be introduced early. Nation-wide lockdown may not further reduce the speed of *R*_*t*_ reduction compared to regional quarantine measures as seen in Italy. In countries where disease transmissions have slowed down, lifting of quarantine measures may lead to a persistent transmission rate delaying control of epidemic and thus should be implemented with caution and close monitoring.

## Data Availability Statement

Publicly available datasets were analyzed in this study. This data can be found here: https://github.com/COVID19BIOSTAT/covid19_prediction.

## Author Contributions

DZ and YW conceived the study. QW and DZ implemented the codes. SX, QW, and YW made figures and tables. All authors interpreted the results, contributed to writing the article, and approved the final version for submission.

## Conflict of Interest

The authors declare that the research was conducted in the absence of any commercial or financial relationships that could be construed as a potential conflict of interest.
