# Supplementary material for: Survival-Convolution Models for Predicting COVID-19 Cases and Assessing Effects of Mitigation Strategies
Source: Front Public Health. 2020 Jul 3;8:325. doi: 10.3389/fpubh.2020.00325 (PMC7347904; doi:10.3389/fpubh.2020.00325)
Supplement: Supplementary file 1 [file Data_Sheet_1.pdf]

Supplementary Material for "Survival-Convolution Models for Predicting COVID-19 Cases and Assessing Effects of Mitigation Strategies"

Qinxia Wang[1], Shanghong Xie[1], Yuanjia Wang[1,*], Donglin Zeng[2,*]

[1] Department of Biostatistics, Mailman School of Public Health, Columbia University, New York, NY, USA ;
[2] Department of Biostatistics, Gillings School of Public Health, University of North Carolina at Chapel Hill, Chapel Hill, NC, USA

Correspondence*: Yuanjia Wang and Donglin Zeng
yw2016@cumc.columbia.edu, dzeng@email.unc.edu

June 7, 2020

Model Estimation, Inference, and Updated Results

We propose a parsimonious survival-convolution model for predicting key statistics of COVID-19 epidemics (e.g., daily new cases) and evaluate public health intervention effect. We model the transmission rate $a(t)$ as a non-negative piece-wise linear function (linear spline and assume $a(t) \geq 0$). For China and South Korea, $a(t)$ is given as follows:

$$a(t) = \begin{cases} a_0^+ & t < t_1 \\ (a_0 + a_1(t - t_1))^+ & t \geq t_1 \end{cases}, \tag{s1}$$

where $x^+ = \max(x, 0)$ and t_1 is the calendar time of reporting the first case. That is, before the first case is reported, the public is unaware and the infection is latent, so the transmission rate is assumed to be a constant; however, once the first case is reported, the public is alerted and various response strategies are gradually introduced and take effect, so that we expect the transmission rate will decrease (i.e., $a_1 \leq 0$). In this simple model, there are three parameters that will be estimated from data, including t_0 (the date of the first case), a_0, and a_1.

When a massive public health intervention (e.g., nation-wide lockdown) is introduced at some particular date, we further add an additional linear function after this date and

introduce a new slope parameter. Thus, the difference in the rate of change in $a(t)$ before and after an intervention reflects its effect on reducing disease transmission (i.e., "flattening the curve"). Furthermore, since the intervention effect may diminish over time, we introduce slope parameters two weeks after the intervention (considering the incubation period as 14 days) to capture the longer-term effect. Therefore, for Italy and US we place additional knots at t_2 (the date of national lockdown for Italy and the declaration of national emergency for US) and t_3 (two weeks after t_2). The transmission rate is modeled as:

$$a(t) = \begin{cases} a_0^+ & t < t_1, \\ (a_0 + a_1(t - t_1))^+ & t_1 \leq t < t_2, \\ (a_0 + a_1(t_2 - t_1) + a_2(t - t_2))^+ & t_2 \leq t < t_3, \\ (a_0 + a_1(t_2 - t_1) + a_2(t_3 - t_2) + a_3(t - t_3))^+ & t \geq t_3. \end{cases} \tag{s2}$$

A long observational period is available for Italy. We place another knot four weeks after t_2 to capture potential long-term effect of the intervention.

Let θ denote all parameters in the transmission rate $a(t)$ (e.g, $a_0, \cdots, a_k$ in equations s1 and s2) and t_0. We divide the reported daily new cases into training data for estimating parameters and testing data for validation. Denote by $Y_o(t_1), Y_o(t_1+1), Y_o(t_1+2), ..., Y_o(t_2)$, the training data consisting of the daily new cases reported from the date of the first reported case, t_1, to the last date in the training set, t_2. To estimate θ using the training data, first note that the number of daily confirmed tested positive cases is a measure of the number of infected cases out of transmission due to a positive COVID test (i.e., $Y(t)$) observed with error (e.g., reporting error, tested positive but not practicing social distancing). Second, it is plausible that the error variability is proportional to the underlying true number of cases (e.g., holds for Poisson random variables). Our model is $Y_o(t) = Y(t) + \sqrt{Y(t)}\epsilon(t)$, where $\epsilon(t)$ represents a residual term. Let $Y(t;\theta)$ denote the predicted new case number at day t for a given θ using recursive equations in (1) and (2) in the main manuscript. We minimize the following loss under a square-root transformation

$$\sum_{t_1 \leq t \leq t_2} \left[\sqrt{Y_o(t)} - \sqrt{Y(t;\theta)} \right]^2 \tag{s3}$$

to estimate θ. The square-root transformation is applied to the daily cases since it is a variance stabilizing transformation for Poisson counts. Computationally, we perform a grid search to estimate t_0. For each t_0, we apply a gradient-based optimizer with adaptive learning rate (i.e., *Adam*[1]) to obtain other parameters. The algorithm is implemented in Tensorflow[2]. We let $\hat{\theta}$ be the minimizer of (s3). With $\hat{\theta}$, we can use equations (1) and (2) in the main manuscript to predict any new daily cases in future dates. Furthermore, by

comparing the estimated $a(t)$ (and correspondingly, R_t) before and after a public health intervention is implemented, we can estimate the intervention effect in terms of the change of transmission rates under the longitudinal pre- and post-intervention design.

For statistical inference such as obtaining confidence intervals of predicted numbers or estimated intervention effects, we assume that the standardized residuals, $[Y_o(t) - Y(t;\theta)] / \sqrt{Y(t;\theta)}$, are exchangeable. Thus, permutation method can be used. We permute the estimated residuals and reconstruct observed cases by adding permuted residuals multiplied by the square-root of the observed case numbers. We repeat this process 500 times and re-analyze each set of permuted data to yield a set of estimates for θ, the corresponding set of predictions for $Y(t;\theta)$ and estimated intervention effects. We obtain 95% confidence intervals using empirical quantiles of the estimates under permutation.

To model the distribution of time to symptom onset since infection, we use the existing knowledge of SARS-CoV-2 virus incubation period. Previous work[3] indicates that the incubation period for SARS-CoV-2 has an average of 5.2 days, and the longest time to symptom onset since infection was reported up to 21 days. Thus, we model the survival function of presenting COVID-19 symptoms as an exponential distribution with a mean of 5.2 truncated at 21, and use this distribution to approximate $S(m)$ in equations (1) and (2) in the main manuscript. In a set of sensitivity analyses, we examine the influence of using a longer mean parameter of this distribution. For the sensitivity analysis of the US, we use a mean value of $5.2 + 4 = 9.2$ (an average of 4-day lag between symptom onset and reporting of daily new cases was observed in a CDC report[4]). For the sensitivity analysis of Italy, we use a mean value of $5.2 + 5.3 = 10.5$ days (an average of 5.3-day lag between symptom onset and reporting of daily new cases was observed in Italy[5]). The results in Figure S2 show that the fitted curves of daily new cases under different parameters of $S(m)$ are identical for US. For Italy, the fitted curves over training data period are almost identical and there is a slight difference at the tail (Figure S3).

We update the analysis of US epidemic using more training data from Feb 21 to May 29. The knots are placed on March 13 (national emergency) and every two weeks (length of incubation period) after that until April 24 to account for potential changes in the transmission rates. We leave 5 weeks of training data before May 29 to robustly determine the trend of the transmission rate for future predictions. The observed training and testing data (May 30 to June 6) are plotted in Figure S4A. With 500 permutation samples, the 95% confidence interval is included for the testing data after May 30. The effective reproduction number R_t is calculated using the piecewise transmission rate and plotted in Figure S4B. Similar to Figure 3B in the main manuscript, the R_t decreases at a faster rate after the declaration of national emergency on March 13, but slows down

when R_t is closer to 1.0. Recently, R_t is near a constant between 1.1 and 1.2 without a clear evidence of decreasing. Although there is a chance to expect less than 100 daily new cases by November 8 this year (with a predicted total number of cases as 2,714,972), the confidence interval suggests some possibility that the daily cases will start to increase again. In fact, some states have experienced an increasing trend in daily new cases since re-opening (e.g., California, Texas, North Carolina). Given recent data, we can see that the US is still in the midst of the epidemic by June 7, 2020, and careful mitigation measures should be maintained to prevent an uptake in daily new cases and another outbreak.

References

1 Kingma DP, Ba J. Adam: A method for stochastic optimization. *arXiv preprint* **arXiv:1412.6980** (2014).

2 Abadi M, Agarwal A, Barham P, et al. TensorFlow: large-scale machine learning on heterogeneous systems (2015). Software available from https://www.tensorflow.org.

3 Li Q, Guan X, Wu P, Wang X, Zhou L, Tong Y, et al. Early transmission dynamics in Wuhan, China, of novel coronavirus–infected pneumonia. *New England Journal of Medicine* **382** (2020) 1199–1207.

4 Centers for Disease Control. Characteristics of health care personnel with COVID-19 — United States, February 12–April 9, 2020. *Morbidity and Mortality Weekly Report* **69** (2020) 477–481. doi:10.15585/mmwr.mm6915e6.

5 Riccardo F, Ajelli M, Andrianou X, Bella A, Del Manso M, Fabiani M, et al. Epidemiological characteristics of COVID-19 cases in Italy and estimates of the reproductive numbers one month into the epidemic. *medRxiv* (2020). doi:10.1101/2020.04.08.20056861.

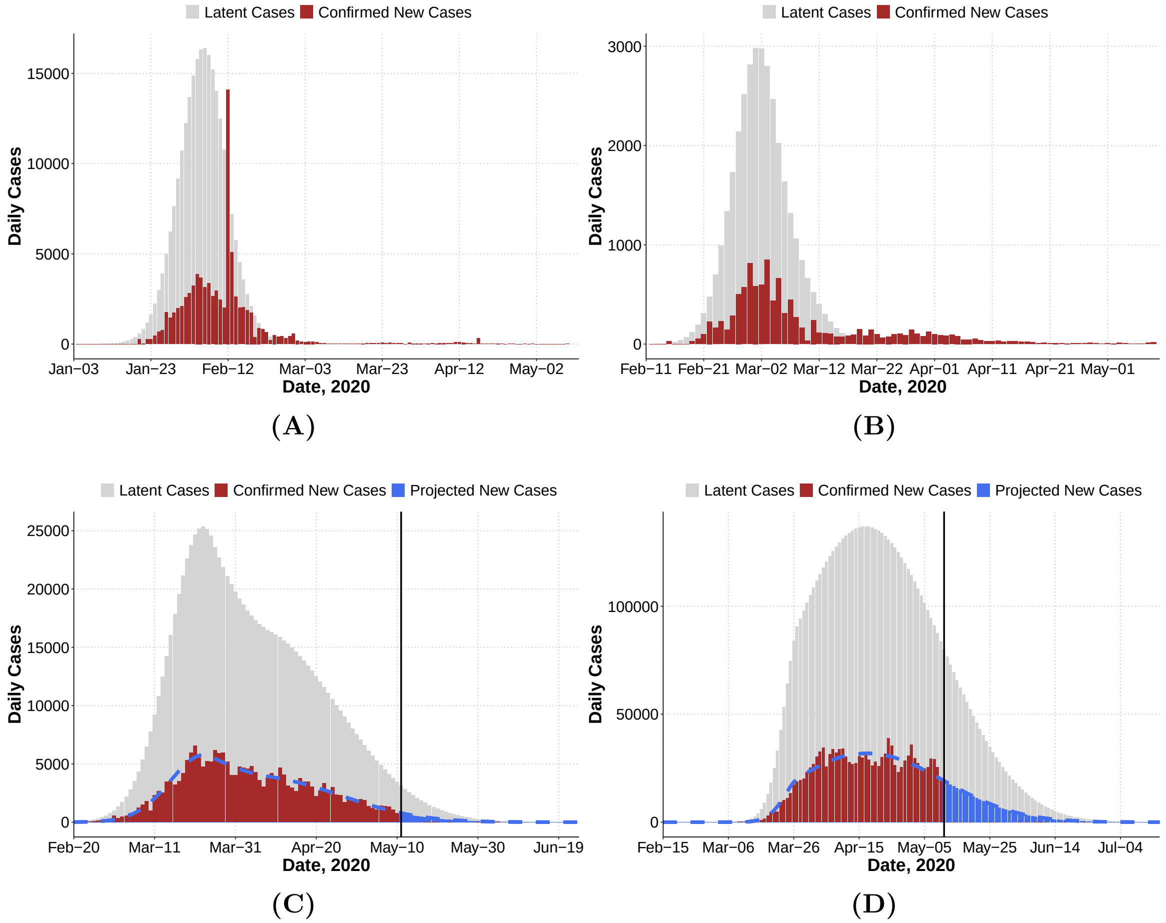

Figure S1: Latent and confirmed cases on each day in each country. Number of latent cases on day t (i.e., estimated $M(t) - Y(t)$) includes all pre-symptomatic cases infected k days before but have not been detected by day t. Solid lines separate observed number of cases and predicted number of cases. **(A)** China. **(B)** South Korea. **(C)** Italy. **(D)** United States.

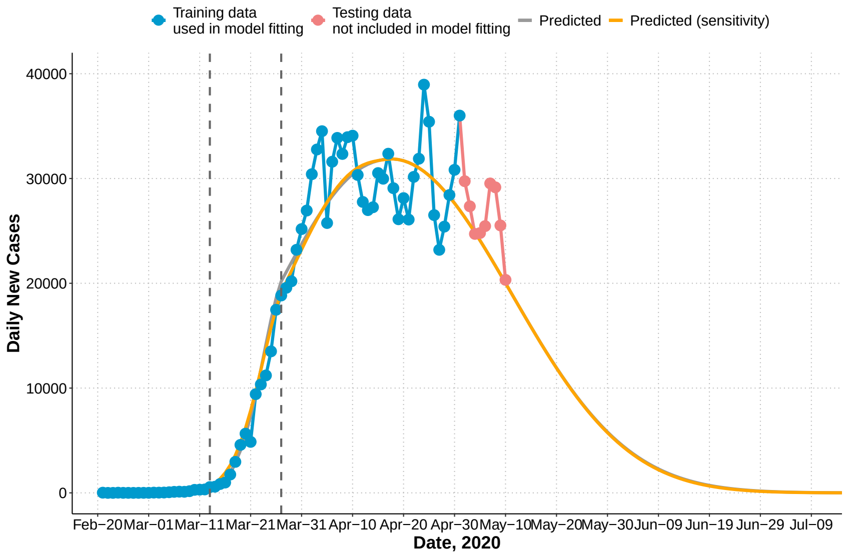

Figure S2: Sensitivity analysis of the US. Observed and predicted daily new cases comparing using an exponential distribution with a mean of 5.2 (grey) and with a mean of 9.2 (orange). First dashed line indicates the declaration of national emergency (March 13). Second dashed line indicates two weeks after (March 27). Training data: February 21 to May 1; Testing data: May 2 to May 10. Fitted curves under different parameters of $S(m)$ are nearly identical.

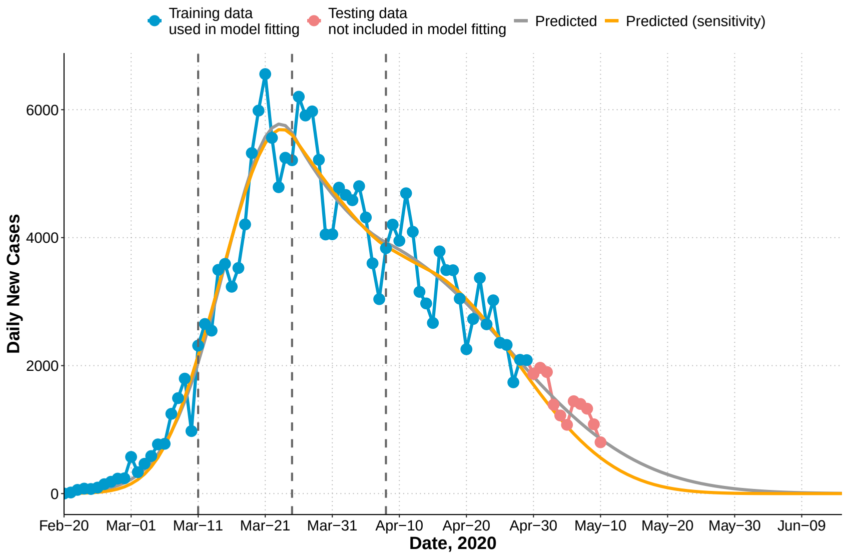

Figure S3: Sensitivity analysis of Italy. Observed and predicted daily new cases comparing using an exponential distribution with a mean of 5.2 (grey) and with a mean of 10.5 (orange). First dashed line indicates the national lockdown (March 11). Second and third dashed lines indicate two weeks after. Training data: February 20 to April 29; Testing data: April 30 to May 10. Fitted curves under different parameters of $S(m)$ are similar.

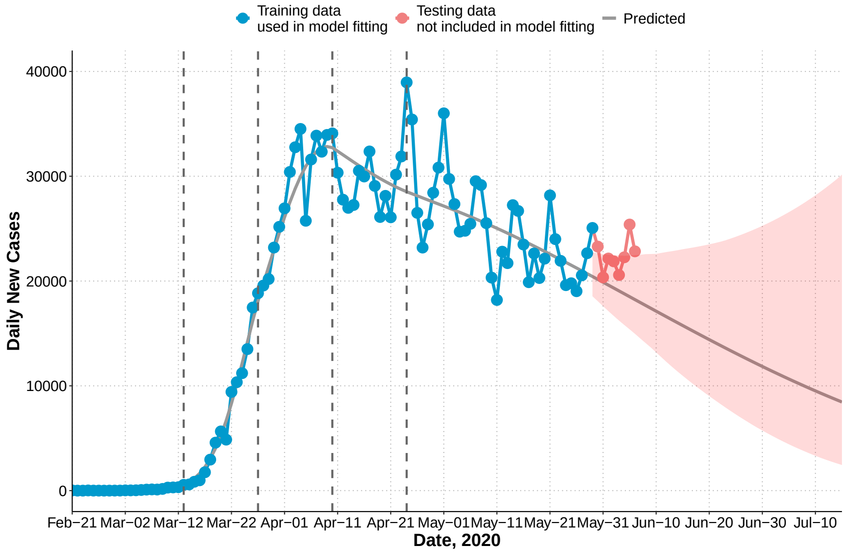

(A)

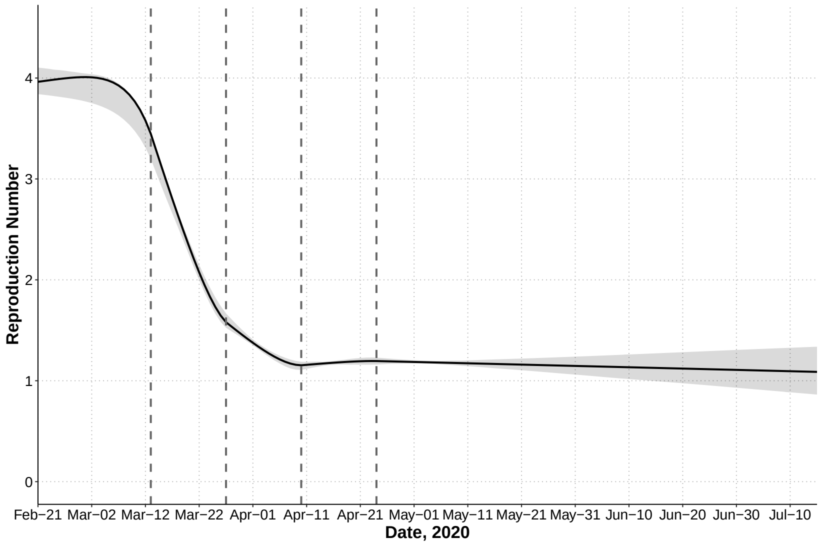

(B)

Figure S4: United States: observed and predicted daily new cases, 95% confidence intervals. First dashed line indicates the declaration of national emergency (March 13). The second to fourth dashed lines indicate every two weeks after (March 27). Training data: February 21 to May 29 (11 weeks after declaring national emergency); Testing data: May 30 to June 6. **(A)** Observed and predicted daily new cases. **(B)** Effective reproduction number R_t.
